# The relationship between adolescents' swimming self-efficacy and high-risk behaviors: a chain mediating model based on latent profile analysis

**DOI:** 10.3389/fpubh.2026.1869352

**Published:** 2026-08-21

**Authors:** Xi Yang, Shujie Xiang, Hui Zhang, Chao Xie

**Affiliations:** School of Physical Education, Hubei Minzu University, Enshi, China

**Keywords:** high-risk swimming behaviors, latent profile analysis, swimming self-efficacy, water safety knowledge, water safety skills

## Abstract

**Background:**

Drowning is the leading cause of injury-related deaths among teenagers in our country. High-risk swimming behaviors show strong correlational linkage with adolescent drowning incidents.

**Methods:**

A survey was conducted with 3,239 teenagers with an average age of 12.73 ± 1.54. The swimming self-efficacy scale, water safety knowledge scale, water safety skills scale, and swimming high-risk behavior scale were used. The latent profile analysis was employed to explore differences in high-risk swimming behaviors across subgroups of swimming self-efficacy, and a chain mediation model was constructed.

**Results:**

The results indicate that swimming self-efficacy comprises four latent categories: fear-avoidant Swimmer (58.6%), Tentative Swimmer (20.7%), Assertive Swimmer (14.9%), and Overconfident Swimmer (5.8%). High-risk swimming behaviors increased sequentially across profiles (all *P* < 0.001). Compared with the Fear-Avoidant Swimmer, the Tentative Swimmer [β = 0.005, 95%CI (0.002, 0.008), *P* < 0.001] and the Assertive Swimmer [β = 0.006, 95%CI (0.004, 0.010), *P* < 0.001] exerted statistically significant chain-mediated effects on high-risk swimming behaviors through water safety knowledge and skills. In contrast, the chain mediation effect did not hold for Overconfident Swimmer [95% CI (−0.001, 0.008)]; water safety skills alone had a statistically significant mediating effect on high-risk swimming behaviors [β = 0.036, 95% CI (0.021, 0.055), *P* < 0.001].

**Conclusion:**

The model indicates that adolescents exhibit group heterogeneity in swimming self-efficacy, which is characterized by a dual association with high-risk swimming behaviors: as self-efficacy increases, the frequency of high-risk behaviors also rises. However, groups with high swimming self-efficacy that are supported by water safety knowledge and skills can reduce the risk of injury due to their comprehensive safety preparedness. In contrast, excessive self-efficacy not grounded in cognitive and skill-based support is prone to inducing reckless risk-taking behavior. At the same time, a bias-corrected self-help method mediation analysis revealed that, within this mediation chain, the mediating effects of “Tentative swimmer” and “Assertive swimmer” were relatively weak. This suggests that the risk-mitigating effects of traditional, comprehensive water safety education—which relies on a chain of knowledge and skills—are very limited. Implementing stratified interventions targeting adolescents' swimming self-efficacy is a crucial measure for reducing the risk of drowning.

## 1 Introduction

Drowning has become a significant public health issue globally, causing substantial casualties among adolescents (1, 2). The 2022 China Youth Drowning Prevention Big Data Report (2) reveals that drowning is the leading cause of death among Chinese children and adolescents aged 0–17. Adolescents account for the largest proportion of drowning case. Despite adolescents' cognitive development, their safety awareness remains relatively weak, their ability to assess Water risks is insufficient, and they are prone to making dangerous decisions (3, 4), making them a high-risk group for drowning. Existing research indicates that examining high-risk swimming behaviors is a crucial antecedent variable in explaining adolescent drowning (5, 6). High-risk swimming behaviors refer to actions taken by individuals or groups in Water environments that threaten their own or others' health and safety. Typical manifestations include: ignoring water warning signs and management rules, overestimating one's swimming ability and tolerance for risk, and lacking self-rescue and mutual aid skills in drowning situations (7). Such behaviors not only directly endanger individuals and others but also pose severe challenges to public health management and safety education. Research indicates that enhancing Water safety knowledge and skills effectively guides individuals toward safer behaviors and reduces risk decision biases (8), while increased self-efficacy boosts behavioral engagement (9). Therefore, this study will explore how protective factors that inhibit high-risk swimming behaviors among adolescents play a role in the association between swimming self-efficacy and high-risk behaviors.

Self-efficacy refers to an individual's belief in their ability to successfully perform a specific behavior or achieve a particular goal in each situation. Bandura A. noted that varying levels of belief correspond to distinct behavioral patterns (9). Low self-efficacy tends to correspond to behavioral avoidance, abandonment of attempts, or anxiety (10). Conversely, moderate self-efficacy enhances motivation, persistence, and effort, making individuals more inclined to embrace challenges and persevere in the face of difficulties (11, 12). Meanwhile, Singh (13) and Dassa et al. (14) suggest that maladaptive, excessively high self-efficacy may trigger overconfidence, causing individuals to overestimate success probabilities and underestimate potential risks. Given the non-linear correlation of self-efficacy on behavior, this study hypothesizes that the role of swimming self-efficacy in swimming-related high-risk behaviors may similarly exhibit non-linear characteristics. Regarding this issue, our research team discovered through systematic outdoor observation and data collection that outdoor wild swimming enthusiasts and lifeguards, who frequently perform high-difficulty swimming maneuvers or water rescues, exhibit extremely low drowning rates. Conversely, certain groups possessing basic swimming skills and awareness of hidden risks show relatively high drowning rates. Does this striking group disparity have an intrinsic connection to the level or type of swimming self-efficacy? That is, might different levels or types of swimming self-efficacy correlate with distinct patterns of high-risk swimming behaviors among adolescents?

### 1.1 The relationship between the latent profile of swimming self-efficacy and high-risk behaviors

To explore such potential group heterogeneity, this study employs individual-centered latent profile analysis (LPA) to investigate variable heterogeneity. The advantage of LPA lies in its ability to identify latent subgroups with similar characteristics (e.g., distinct patterns of swimming self-efficacy) based on objective, rigorous model fit indices, while estimating the probability of individual membership across types. This approach addresses the limitations of relying solely on formal scale classifications (15). Based on the theoretical background, methodological considerations, and empirical evidence, this study proposes the following hypotheses: H1: significant group heterogeneity exists in adolescent swimming self-efficacy, identifiable through LPA into distinct latent categories; and the mediating patterns of different swimming self-efficacy categories on adolescent high-risk swimming behaviors exhibit significant differences.

### 1.2 The mediating role of water safety knowledge and skills

Water safety knowledge (16) and water safety skills (17) are the key variables influencing the safety of teenagers in water environments. The former aims to help teenagers establish correct concepts of water safety and enhance their individual risk perception, thereby reducing the incidence of risky behaviors (18); based on this, this study proposes Hypothesis H2: water safety knowledge plays a mediating role between the potential category of swimming self-efficacy and high-risk swimming behaviors. Water safety skills refer to an individual's ability to prevent accidents, respond to emergencies, and ensure their own and others' safety in water environments, covering procedural knowledge such as swimming safety skills, self-rescue skills, drowning rescue skills, and first aid skills (19). Empirical research has shown that self-efficacy can positively correlate with learners' acquisition of safety knowledge and the practice of skills (20); therefore, this study further proposes Hypothesis H3: water safety skills play a mediating role between the potential category of swimming self-efficacy and high-risk swimming behaviors.

### 1.3 The chain-like mediating role of water safety knowledge and skills

Further analysis indicates that knowledge and skills are not isolated from one another. Pavese and Beddor (21) contend that skills are essentially “knowledge of how to perform actions appropriately in practice.” Their development relies on the recognition of action intent and method, which are inextricably linked. Specifically, water safety knowledge forms the cognitive foundation for water safety skills: young people must first understand a series of safety rules before they can transform this knowledge into the ability to avoid dangers and perform emergency self-rescue in actual water environments. Simultaneously, the proficient application of Water safety skills reinforces understanding and retention of Water safety knowledge, creating a bidirectional promotion between cognition and behavior. Empirical research confirms that individuals with different self-efficacy levels exhibit variations in their willingness to learn Water safety knowledge and their mastery of Water safety skills. Those with high self-efficacy are more inclined to proactively learn safety knowledge, and sufficient knowledge reserves enable them to acquire skills more efficiently (22). Furthermore, the efficiency of converting Water safety knowledge into skills has been shown to directly correlation the frequency of high-risk behaviors (23). Based on the intrinsic connection between knowledge and skills and the empirical evidence supporting it, this study proposes the following hypothesis: H4. Water safety knowledge and water safety skills play a chain mediating role in the relationship between swimming self-efficacy latent classes and high-risk swimming behavior.

Therefore, this study aims to comprehensively consider the causes of high-risk swimming behavior and adopt latent profile analysis (LPA) to classify the latent classes of adolescents' swimming self-efficacy. It intends to explore whether there are differences in the level of high-risk swimming behavior among adolescents with different latent classes of swimming self-efficacy, and to test the mediating hypotheses (H2, H3, H4). The findings are expected to provide valuable insights for public health practices aimed at reducing adolescent drowning risks and safeguarding their physical and mental wellbeing.

## 2 Research methods

### 2.1 Common method bias test

The Harman one-factor test was used to calculate and test all items. The results showed that there were a total of six factors with eigenvalues greater than 1. The first principal component obtained before rotation accounted for 23.993% of the total factor loadings, which did not exceed the critical threshold of 40%. This indicates that there are no serious common method bias issues in the questionnaire survey results of this study.

### 2.2 Participants

Using random cluster sampling, questionnaires were administered to 3,600 students in grades 5–9 across Hubei Province. Prior to formal administration, the research team obtained informed consent from the education bureau, school administrators, homeroom teachers, parents, and the students themselves. The assessment was organized by class to ensure efficiency and order. All questionnaires were completed anonymously, collecting demographic information including gender, age, and grade level. After detailed instructions, all participants completed the questionnaire within 45 min. After excluding invalid responses, 3,239 valid questionnaires (87.38%) were collected and processed. The average age of participants was 12.73 ± 1.54 years, comprising 1,713 males (52.89%) and 1,526 females (47.11%). This study was approved by the Ethics Committee [(2024) No. 101].

### 2.3 Research tools

#### 2.3.1 Swimming self-efficacy scale

The Swimming Self-Efficacy Scale developed by Theodorakis (24) was employed. This scale comprises six items, focusing on assessing participants' self-efficacy expectations regarding completing progressively longer swimming distances within 20 s. Scores ranged from 1 to 10 (1 = not at all, 10 = very certain). The sum of all item scores constituted the participant's total self-efficacy score, with higher scores indicating stronger perceived self-efficacy regarding their Water safety skills. The scale demonstrated good reliability and validity, with a Cronbach's alpha coefficient of 0.85.

#### 2.3.2 Water safety skills and knowledge scale

The Student Water Safety Knowledge, Attitude, and Behavior Scale developed by Xia Wen (25) was adopted for this study. The water safety skills and knowledge sub-questionnaire, comprising 19 items, was selected as the measurement tool. Results from confirmatory factor analysis indicated good model fit indices (χ^2^/df = 3.06, CFI = 0.86, NFI = 0.92, IFI = 0.94, RMSEA = 0.055), indicating strong internal consistency for the entire scale. The Cronbach's α coefficients for the Water safety skills and knowledge subscales were 0.964 and 0.963, respectively, demonstrating good internal consistency validity and reliable reliability and validity. All items in the scale employ a 5-point Likert scale (1 = Not at all familiar, 5 = Very familiar). Higher scores indicate greater skill and knowledge levels. Furthermore, the scale is closely aligned with China's Water safety culture and the actual circumstances of children and adolescents, enhancing the questionnaire's applicability and comprehensibility.

#### 2.3.3 High-risk swimming behavior scale

The Adolescent High-Risk Swimming Behavior Scale developed by the research team was employed (26)^.^ This scale comprises four dimensions: attitudinal errors, mistakes, general violations, and aggressive violations, encompassing a total of 20 items. It explained 68.444% of the total variance. Confirmatory factor analysis results indicated good model fit indices (χ^2^/df = 3.672, CFI = 0.938, NFI = 0.952, TLI = 0.957, IFI = 0.938, RMSEA = 0.045). The Cronbach's α coefficient for the scale was 0.859, indicating good internal consistency validity and demonstrating sound reliability and validity. All items employed a 5-point Likert scale, requiring participants to rate each indicator on a 1–5 scale, where 1 signifies very poor and 5 signifies very good. A lower score indicates that the individual engages in high-risk swimming behaviors less frequently, while a higher score suggests that the occurrence frequency of such high-risk behaviors is higher for the individual.

### 2.4 Data processing

This study employed SPSS 27.0 for descriptive statistical analysis and correlation analysis to reveal the net effect of the predictor variables on the outcome variable. Mplus 8.3 was used to conduct individual-centered latent profile analysis (LPA), dividing homogeneous adolescent subgroups based on the 6 items of the swimming self-efficacy scale. Among them, the measurement data are presented as the mean ± standard deviation ($\overset{-}{X}\pm S$). Sequentially, 1–5 type profile models were constructed, and the optimal model was selected by combining multiple fit indicators: smaller AIC, BIC, and aBIC values indicated better model fit; an entropy value of Entropy >0.9 indicated high group accuracy; LMRT and BLRT tests had significant *p*-values. The entropy value of the four-category model was 0.965, and both tests were significant. The LMRT test for the five-category model was not statistically significant. Therefore, the 4 profiles were determined as the optimal solution. Finally, the PROCESS 4.1 developed by Hayes was used for category mediating effect analysis (27). Compared with traditional variable-centered regression analysis, LPA can capture the heterogeneous grouping characteristics of swimming self-efficacy within the sample.

## 3 Results

### 3.1 Correlation analysis of variables

As shown in Table 1, high-risk swimming behaviors are significantly positively correlated with Water safety skills and swimming self-efficacy, and significantly negatively correlated with Water safety knowledge.

**Table 1:** Descriptive statistics and correlation analysis.

Variables	Mean ±SD	1	2	3	4
1. Water safety knowledge	3.509 ± 1.080	1			
2. Water safety skills	2.408 ± 1.180	0.427^**^	1		
3. Swimming self-efficacy	2.804 ± 2.274	0.100^**^	0.332^**^	1	
4. High-risk swimming behavior	1.523 ± 0.542	−0.118^**^	0.103^**^	0.252^**^	1

^**^p < 0.01.

### 3.2 Latent profile analysis of adolescents' swimming self-efficacy

Using the six items of the Self-Efficacy Scale as independent variables, latent profile models with 1–5 categories were constructed. The results (Table 2) show that as the number of categories increased, the AIC, BIC, and aBIC values gradually decreased, indicating progressively optimized model fit. The entropy values for all categories were greater than 0.90, indicating good classification accuracy for each model. The BLRT p-values were statistically significant for all models. The LMRT *p*-values were statistically significant for the 1- to 4-category models, while the LMRT *p*-value for the 5-category model was not statistically significant. This indicates that the 4-category model is the optimal model.

**Table 2:** Fitting information for potential profile analysis of students' self-efficacy.

Model	AIC	BIC	aBIC	Entropy	LMRT (*P*)	BLRT (*P*)	Category probability
1	91,604.456	91,677.452	91,639.323				1
2	77,639.750	77,755.327	77,694.956	0.959	< 0.001	< 0.001	0.762/0.238
3	71,186.557	71,344.715	71,262.102	0.968	< 0.05	< 0.001	0.652/0.085/0.263
4	67,829.808	68,030.548	67,925.692	0.965	< 0.05	< 0.001	0.586/0.207/0.149/0.058
5	66,029.304	66,272.625	66,145.528	0.968	0.4758	< 0.001	0.586/0.184/0.058/0.118/0.054

As shown in Table 3, in the four-classification model, the correct classification probability for the first category is 99.3%, for the second category is 94.0%, for the third category is 98.1%, and for the fourth category is 99.3%. This indicates that the latent profile analysis results in this study are reliable. The average posterior probabilities for all four profile categories exceeded 90%, and the vast majority of participants could be accurately assigned to their corresponding subgroups, with no significant cross-classification errors.

**Table 3:** Average posterior probability of each section.

Category	1	2	3	4
1	0.993	0.007	0	0
2	0	0.015	0.981	0.004
3	0.043	0.940	0.016	0
4	0	0	0.007	0.993

**Figure 1:**
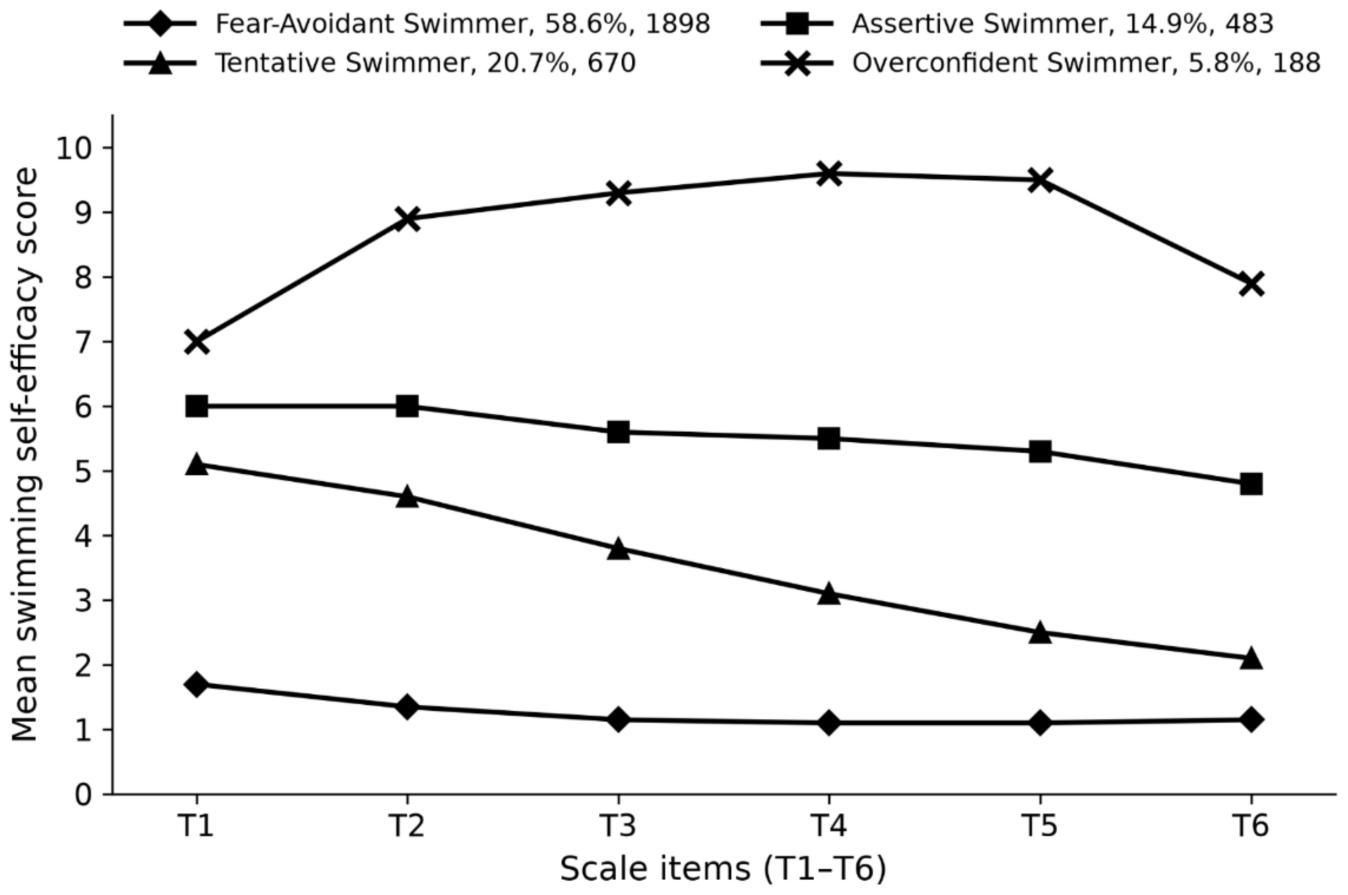
The average scores of the four categories of students on each item of the swimming self-efficacy scale. On the X-axis, T1–T6 represent the six items of the swimming self-efficacy scale; the Y-axis represents the “average swimming self-efficacy score”.

Based on the number of adolescents across each indicator and their scores on the six items of the Swimming Self-Efficacy Scale (see Figure 1), adolescent swimming self-efficacy was categorized into four levels: low self-efficacy C1 (1,898 individuals), moderate self-efficacy C2 (670 individuals), high self-efficacy C3 (483 individuals), and excessive self-efficacy C4 (188 individuals).

Based on Table 4 and Figure 1, the naming of the four profile categories was based on a comprehensive analysis of differences among the three sets of variables—self-efficacy item score curves, safety knowledge, and skills, and high-risk behaviors—rather than relying solely on the total self-efficacy score. the four latent categories were named as follows: category 1 exhibited the lowest scores in swimming self-efficacy, with the lowest scores in water safety knowledge (3.42 ± 1.14), water safety skills (2.07 ± 1.12), and high-risk swimming behaviors (1.43 ± 0.43), indicating insufficient knowledge and skill mastery and reluctance to attempt swimming. Thus, C1 is defined as the “Fear-Avoidant Swimmer” Category 2 exhibits moderate swimming self-efficacy, with water safety knowledge (3.62 ± 0.93), Water safety skills (2.83 ± 1.02), and high-risk swimming behaviors (1.59 ± 0.50). This indicates that individuals with moderate self-efficacy (C2) possess preliminary confidence in short-distance swimming tasks but experience a rapid decline in self-efficacy for medium-to-long-distance tasks, exhibiting cautious behavior. Thus, C2 is defined as the “Tentative Swimmer” Category 3 maintained consistently high swimming self-efficacy across all items despite slight declines in mean scores, demonstrating balanced self-assurance for tasks of varying difficulty. Scores for Water safety knowledge (3.69 ± 0.92), Water safety skills (3.01 ± 1.06), and swimming risk behaviors (1.68 ± 0.62) were all at high levels, indicating that these individuals possess good knowledge and skill mastery with moderate behavioral tendencies. Therefore, Group C3 is defined as the “Assertive Swimmer” Category 4 had the highest swimming self-efficacy, with water safety knowledge (3.5 ± 1.29) and Water safety skills (2.79 ± 1.46) were at moderate levels, while swimming risk-taking behaviors (1.86±1.05) scored highest. This indicates a mismatch between knowledge, ability, and behavior within this category. Individuals exhibit strong confidence in their swimming abilities coupled with overconfidence, reflecting excessively high swimming self-efficacy. Therefore, Group C4 is defined as the “Overconfident Swimmer” (High Self-Efficacy Mismatch Subgroup).

**Table 4:** The mean and standard deviation of different categories of students' swimming self-efficacy in each dimension ($\overset{-}{X}\pm S$).

Variable	C1 fear-avoidant swimmer (*n* = 1,898)	C2 tentative swimmer (*n* = 670)	C3 assertive swimmer (*n* = 483)	C4 overconfident swimmer (*n* = 188)	*F*	η2	Post-event comparison
Water safety knowledge	3.419 **±** 1.135	3.616 **±** 0.930	3.690 **±** 0.920	3.509 **±** 1.287	11.31^***^	0.01	C1 < C4 < C2 < C3
Water safety skills	2.067 **±** 1.120	2.834 **±** 1.017	3.006 **±** 1.060	2.787 **±** 1.461	147.17^***^	0.12	C1 < C4 < C2 < C3
High-risk swimming behavior	1.426 **±** 0.426	1.592 **±** 0.496	1.675 **±** 0.620	1.855 **±** 1.046	63.54^***^	0.06	C1 < C2 < C3 < C4

^***^p < 0.001.

### 3.3 Comparison of scores across dimensions for four categories of adolescents

Statistically significant differences in mean scores were observed across different adolescent swimming self-efficacy categories on the High Risk Swimming Behavior Scale, Water Safety Skills Scale, and Water Safety Knowledge Scale (see Table 4). *Post-hoc* multiple comparisons revealed that C1 scored significantly lower than C2, C3, and C4 on water safety knowledge, water safety skills, and swimming risky behavior. C2 showed no significant difference in Water safety knowledge compared to C3 and C4, but scored significantly lower than C3 in Water safety skills and significantly lower than C3 and C4 in swimming risk behaviors; C3 demonstrated the highest levels in both Water safety knowledge and Water safety skills, with Water safety skills significantly higher than C1, C2, and C4, but swimming risk behaviors higher than C1 and C2 and lower than C4; C4 exhibits moderate Water safety knowledge comparable to C2 and C3, Water safety skills below C2 and significantly below C3, but significantly higher swimming risk behaviors than other categories.

### 3.4 The mediating role of water safety knowledge and skills

**Figure 2:**
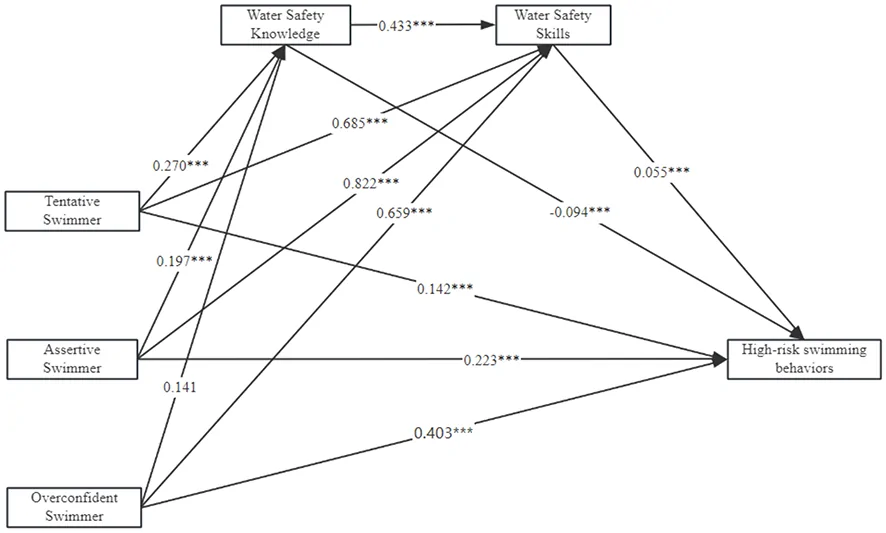
The path model for testing the mediating effect. This model uses “fear-avoidant swimmers” as the reference group for dummy coding; all paths represent unstandardized regression coefficients β, and the analysis controls for age, gender, and grade level. ***p < 0.001.

Using PROCESS 4.1, latent categories of adolescent swimming self-efficacy were virtually coded. Age, gender, and grade level were incorporated as control variables. A chained mediation model was established with the mean Water safety knowledge score and mean Water safety skill score as mediating variables, and high-risk swimming behaviors as the dependent variable. The results showed that the overall total effect was statistically significant (*F* = 63.535, *P* < 0.001). None of the four direct relative effects were zero. The overall direct effect was also statistically significant (*F* = 49.606, *P* < 0.001). According to the effect size evaluation criteria, η2 < 0.01 is considered a small effect, 0.01–0.06 is a medium effect, and >0.06 is a large effect. The differences among groups exhibiting high-risk swimming behaviors were characterized by an η^2^ value of 0.06, indicating a moderate effect size. This suggests that the swimming self-efficacy latent variable can effectively distinguish adolescents with varying levels of high-risk behavior, providing empirical support for the implementation of targeted and precise drowning prevention education. Therefore, a relative mediation analysis was further conducted. Relative mediation analysis revealed (Figure 2): specifically, with the fear-type swimmers as the reference group, Water safety knowledge mediated the relationship between Tentative Swimmer [95%CI (−0.029, −0.009)] and Assertive Swimmer [95%CI (−0.038, −0.015)] and high-risk swimming behaviors, with mediation effect values of −0.019 and −0.025, respectively. while the mediating effect of Water safety knowledge on swimming risk-taking behavior was not significant. Water safety skills mediated the relationship between Tentative Swimmer [95%CI (0.023, 0.052)] and swimming risk-taking behavior, 0.052, Assertive Swimmer [95%CI (0.028, 0.063)], and Overconfident Swimmer [95%CI (0.021, 0.055)] and swimming-risky behaviors, with mediating effect values of 0.037, 0.045, and 0.036, respectively.

Additionally, Water safety knowledge and Water safety skills exhibited chain mediation effects between Tentative Swimmers [95%CI (0.002, 0.008)], Assertive Swimmers [95%CI (0.004, 0.010)], and high-risk swimming behaviors, with effect sizes of 0.013 and 0.012, respectively. However, Water safety knowledge and Water safety skills did not mediate the relationship between overconfident swimmers [95%CI (−0.001, 0.008)] and high-risk swimming behaviors. Simultaneously, Tentative Swimmers, Assertive Swimmers, and Overconfident Swimmers were all positively associated with the mean score of high-risk swimming behaviors, with statistically significant relative total effects (0.166, 0.249, and 0.429, respectively; all 95%CI excluded zero). See Figure 2 for details.

Bias-corrected bootstrap test results (Table 5) indicate that, with Fear-Avoidant Swimmers as the reference group and age, gender, and grade level statistically controlled, water safety knowledge and water safety skills form a significant chain mediation linking Tentative Swimmers and Assertive Swimmers to high-risk swimming behaviors (both 95% CIs exclude zero). However, this sequential pathway is non-significant for Overconfident Swimmers (the 95% CI spans zero). After accounting for the two sequential mediators, Tentative Swimmers (raw β = 0.142, Std. β = 0.263, *p* < 0.001), Assertive Swimmers (raw β = 0.223, Std. β = 0.412, *p* < 0.001), and Overconfident Swimmers (raw β = 0.403, Std. β = 0.743, *p* < 0.001) all showed statistically significant direct associations with high-risk swimming behaviors. It is worth noting that the partially standardized coefficients of both significant chain mediating pathways are close to zero, representing trivial effect sizes. With a sample of 3,239 adolescents, this study has high statistical power; even weak correlations can reach statistical significance, so *p*-values alone cannot reflect the real predictive magnitude of these sequential pathways. In stark contrast, the direct and total partially standardized effects of the three latent profiles range from moderate to large, suggesting substantially stronger real-world predictive links to high-risk swimming behaviors.

**Table 5:** The test results of the mediating model of self-efficacy.

Mediating path	Unstandardized effect (raw β)	Standardized effect (Std. β)	Boot 95% CI lower limit	Boot 95% CI upper limit
Taking the fear-avoidant swimmer as the reference				
Tentative swimmer → water safety knowledge → water safety skills → high-risk swimming behaviors	0.005	0.009	0.002	0.008
Tentative swimmer → water safety knowledge → high-risk swimming behaviors	−0.019	−0.034	−0.029	−0.009
Tentative swimmer → water safety skills → high-risk swimming behaviors	0.037	0.069	0.023	0.052
Tentative swimmer → high-risk swimming behaviors	0.142	0.263	0.095	0.190
Relative total effect	0.166	0.306	0.119	0.212
Assertive swimmer → water safety knowledge → water safety skills → high-risk swimming behaviors	0.006	0.012	0.004	0.010
Assertive swimmer → water safety knowledge → high-risk swimming behaviors	−0.025	−0.047	−0.038	−0.015
Assertive swimmer → water safety skills → high-risk swimming behaviors	0.045	0.083	0.028	0.063
Assertive swimmer → high-risk swimming behaviors	0.223	0.412	0.169	0.277
Relative total effect	0.249	0.460	0.197	0.302
Overconfident swimmer → water safety knowledge → water safety skills → high-risk swimming behaviors	0.003	0.006	−0.001	0.008
Overconfident swimmer → water safety knowledge → high-risk swimming behaviors	−0.013	−0.024	−0.031	0.005
Overconfident swimmer → water safety skills → high-risk swimming behaviors	0.036	0.066	0.021	0.055
Overconfident swimmer → high-risk swimming behaviors	0.403	0.743	0.324	0.482
Relative total effect	0.429	0.791	0.350	0.508

## 4 Discussion

### 4.1 Heterogeneous latent profile analysis of adolescent swimming self-efficacy

This study employed latent profile analysis (LPA) to identify four latent categories of adolescent swimming self-efficacy: fear-avoidant Swimmer (58.6%), Tentative Swimmer (20.7%), Assertive Swimmer (14.9%), and Overconfident Swimmer (5.8%). Based on the relationship between adolescents' beliefs, cognition, ability, and behavior, it verifies the differential correlation of swimming self-efficacy on high-risk swimming behaviors and its underlying pathways of action.

From the distribution of categories, the Fear-Avoidant Swimmer accounts for 58.6%, representing the mainstream among adolescents. Given China's family and school education, these adolescents develop a perception that water is “dangerous” due to overprotective parents who forbid water exposure. Additionally, school drowning prevention education often focuses solely on theoretical lectures. The absence of swimming skill instruction directly results in weak Water abilities, creating a dual constraint of “lack of swimming skills + excessive avoidance of water.” This leads to low swimming self-efficacy and highly cautious behavior, objectively reducing the likelihood of engaging in high-risk swimming activities. Consequently, once unexpectedly submerged in water, the drowning rate is extremely high. This outcome aligns with Bandura's self-efficacy theory, where low self-efficacy tends to trigger avoidance, anxiety, or goal abandonment (9), thereby reducing attempts at high-risk swimming behaviors. Notably, the high proportion in this category reflects the current reality of inadequate swimming safety education and skill training among adolescents. Survey data from Huizhou City targeting parents provides corroborating evidence: among 137,914 valid responses, 72.62% of parents reported their children cannot swim, 78.12% explicitly prohibit outdoor water activities, and 81.07% stated their children's schools do not offer swimming lessons. These findings collectively reveal a collaborative gap between family protection and school education in cultivating Water safety skills. Compared to the swimming-phobic group, the Tentative Swimmer (20.7%) exhibits moderate self-efficacy, with preliminary cognitive and skill development. While attempting basic swimming movements, they remain highly cautious, tending to “dip their toes in the water” without fully committing. Typical behaviors include a tendency to practice in shallow water and familiar environments, with heavy reliance on instructors and assistive tools, while avoiding attempts beyond their capabilities. This reflects how moderate self-efficacy begins to drive behavior through the alignment of cognition and ability.

Among the high swimming self-efficacy group, the core difference lies in the alignment between cognition and ability. Assertive Swimmer (14.9%) possesses both sufficient water safety knowledge and the strongest Water safety skills, demonstrating a high degree of alignment between these two competencies. Among adolescents, achievement motivation driven by high self-efficacy increases the likelihood of engaging in high-risk swimming behaviors and attempting challenging swimming scenarios; however, the combined support of knowledge and skills can mitigate the negative consequences of such high-risk behaviors. This also supports the view that high self-efficacy can enhance adaptive coping (28). Typical examples include divers attempting high-difficulty maneuvers, water rescue volunteers performing emergency interventions, and open-water swimming enthusiasts tackling challenging scenarios. Such behaviors are grounded in dual safeguards: clear risk awareness and robust personal skills, demonstrating the positive effects of high self-efficacy. In contrast, the Overconfident Swimmer (5.8%) exemplifies the negative effects of self-efficacy. Based on the scale results—specifically, perceived swimming ability (measured by the Swimming Self-Efficacy Scale, reflecting adolescents' subjective confidence in their ability to complete swimming tasks) and objective practical skills in the water (measured by the Water Safety Knowledge and Skills Scale)—we found that although these adolescents were aware of risks in the water, their skill levels were only moderately low skill levels. Their behavioral decision-making reveals two core issues: first, while adolescents develop preliminary independent judgment, cognitive biases persist (29), leading them to equate “risk awareness with risk management capability,” thereby nullifying the protective role of water safety knowledge. Second, this group exhibits a pronounced Dunning-Kruger effect (30), where individuals with low Water safety skills tend to overestimate their actual abilities while lacking the metacognitive capacity to accurately assess their capabilities. This miscalibrated self-perception leads the Overconfident Swimmer subgroup to overestimate personal aquatic capacity and engage in frequent risky swimming, matching the high-risk crowd characterized by partial incomplete safety literacy in national cross-sectional research (31). Meanwhile, this is also fully consistent with the “discrepancy between perceived and actual ability” pattern summarized by Moran and colleagues in their cross-national practical swimming skills test: participants with high self-assessments often failed to complete basic floating and underwater survival tasks, yet underestimated hidden underwater dangers (32); Subsequent empirical research on adults conducted by Stanley et al. (33) further validated this cross-age overestimation bias, demonstrating that individuals with mediocre water survival skills consistently overestimate their aquatic abilities regardless of age—which explains why this subgroup exhibited the highest frequency of high-risk swimming behaviors in our sample. It is precisely this dual extreme—the mistaken belief that knowledge equals skill and the overestimation of skill levels triggering inflated self-efficacy—that further amplifies participation in high-risk swimming behaviors, significantly increasing drowning risks. The disconnection between the subjective perception of swimming self-efficacy and actual water competence aligns with the internationally recognized three-dimensional water competence taxonomy proposed by Stallman et al. (34). This framework divides comprehensive water competence into three interrelated core dimensions: awareness of water risks, actual self-rescue and rescue skills, and an objective and accurate self-assessment of one's own water competence. The overconfident swimmers in this study represent a typical group whose water competence structure is imbalanced, with excessive confidence but not effectively balanced by insufficient safety knowledge and weak practical skills. For instance, the fatal shallow-water diving incident involving a physical education teacher in Ningbo, and reports by Hunan Daily on high-risk wild swimming sites in Changsha, frequently document people and children ignoring warning signs to enter the water. They commonly hold the mindset: “I've been swimming for years; I know where the dangers lie and can handle them.” This aligns with prior research concluding that 10- to 14-year-olds relying solely on swimming skills without corresponding water safety awareness may increase their exposure to water bodies and elevate drowning risks (23). It also echoes findings that when high self-efficacy detaches from actual ability, it may display patterns of overly ambitious goal setting or misallocation of resources, alongside weaker performance correlates (35). From the aquatic physical literacy perspective of the ALFAC Consortium (36), such one-sided imbalance of water competence reflects defects in the psycho-social dimension of adolescent aquatic literacy, which cannot be corrected merely through single swimming skill training.

### 4.2 Differences in the mediating effects of swimming self-efficacy subtypes and their dual association mechanisms

Based on the mediation analysis results, Self-efficacy exhibits a protective association: only among the “Tentative swimmers” and “Assertive swimmers” groups was the mediating effect of water safety knowledge significantly negative and strengthened as self-efficacy increased; self-efficacy can curb high-risk swimming behaviors by enhancing water safety knowledge; At the same time, self-efficacy also exhibits a risk-related association: among the three groups—Tentative, Assertive, and Overconfident swimmers—the mediating effect of water safety skills was consistently and significantly positive; increased self-efficacy drives the development of swimming skills, thereby promoting risky water-related behaviors among adolescents. It is worth noting that the mediating effect of water safety knowledge was not significant only among adolescents exhibiting Overconfident Swimmer; the mediating effect of water safety knowledge was not significant. The chain mediation effect through water safety knowledge and Water safety skills was also not significant. Only the mediating role of Water safety skills was significant. This suggests that when an individual's self-efficacy level is excessively high, water safety knowledge fails to reduce high-risk swimming behaviors; among the overconfident group with a skills-ability mismatch, inflated subjective perceptions offset the mediating effect of safety knowledge, leaving only skills to be associated with behavior. These associational pathways aligns with Schönfeld et al. (28) proposition that high self-efficacy enhances adaptive coping and reduces stress responses, whereas excessive self-efficacy may increase risk-taking behaviors or lead to overestimation of capabilities, thereby amplifying the negative impacts of stress.

At the same time, according to the results of the bias-corrected bootstrap test in this study, the chain mediation path from “water safety knowledge” to “water safety skills” was statistically significant for both “Tentative” and “Assertive” swimmers. It is worth noting that the original, unstandardized chained indirect effects were both very small positive values (0.005 and 0.006, respectively). This small positive composite coefficient stems from two opposing sub-paths that offset each other: water safety knowledge alone produces a protective effect, thereby reducing high-risk swimming behaviors; whereas water safety skills promote high-risk aquatic activities. Along this sequential pathway, the slight risk-increasing effect of skills slightly outweighed the risk-buffering function of knowledge. Although these chain pathways reached statistical significance due to the large sample size (*N* = 3,239), their practical mediating effect was negligible and had virtually no capacity to curb risky behavior. These results indicate that the cognitive-skill sequential chain is not the core pathway linking swimming self-efficacy subtypes to high-risk swimming behaviors. The association between the three non-reference subgroups and high-risk swimming behaviors is primarily mediated through direct pathways. The association between the three non-reference subgroups and high-risk swimming behaviors is primarily mediated through direct pathways; among them, the self-confident subgroup exhibited a standardized direct effect of 0.743 and a total standardized effect as high as 0.791, with no statistically significant association found for the chain mediation pathway in this subgroup. This result indicates that adolescents with imbalanced self-efficacy perceptions are prone to directly engaging in high-risk water-related behaviors, and the continuous buffering mechanism from knowledge to skills is completely ineffective in this subgroup. This trivial practical effect of unified knowledge-skill training fully matches the core viewpoint of the aquatic literacy framework proposed by the ALFAC Consortium (36). The framework criticizes traditional one-size-fits-all aquatic education: simple popularization of knowledge and swimming skills cannot fix teenagers' biased self-assessment of aquatic ability, which explains why the chain mediation pathway loses its inhibitory effect on overconfident adolescents. Consistent with Stallman et al. (34), effective drowning prevention education must simultaneously optimize risk cognition, survival skills and objective self-evaluation, rather than merely relying on cognitive and skill teaching alone. education for adolescents, relying solely on the uniform dissemination of safety knowledge and basic swimming skill training is unlikely to effectively mitigate the propensity for high-risk behaviors across different self-efficacy subtypes through chain mediation mechanisms; the core of stratified interventions should focus on correcting adolescents' differentiated cognitive biases regarding water-related activities, as traditional, single-dimensional knowledge and skill training yields very limited benefits in terms of risk mitigation through chain mediation pathways.

Compared to previous studies that predominantly examined the unilateral effects of self-efficacy, positive aspects—such as high perceived self-efficacy promoting farmers' adoption of safe pesticide practices (37)—have been explored. Conversely, negative aspects have been identified in studies involving entrepreneurs (13) and pre-service teachers (14), indicating that inflated or unhealthily elevated self-efficacy levels can adversely impact career development. Prior water safety mediation research only treated swimming self-efficacy as a continuous single variable and reported overall linear associations between safety knowledge, skill, and risk behavior (38, 39). At the international level, the landmark “Can You Swim?” project conducted by Moran et al. (32) enriched this research. The study systematically distinguished between perceived aquatic competence (self-reported swimming confidence) and objectively tested aquatic survival skills, and confirmed that there was only a moderate correlation between these two measures in a sample of adolescents. Adolescents with limited aquatic skills tend to overestimate their swimming abilities, which has become a key risk factor leading to reckless aquatic behavior. To resolve these discrepancies, this study employs latent class analysis and mediation effect testing. It reveals that the key correlate underlying self-efficacy's “double-edged sword” pattern is the alignment between individual cognition and capability. When high self-efficacy synergizes with sufficient knowledge reserves and solid skill levels, it manifests as positive behaviors (e.g., Assertive Swimmer). Conversely, when high self-efficacy becomes detached from knowledge constraints and capability support, it tends to covary with risk perception biases and behavioral overconfidence, alongside patterns of negative behaviors (e.g., Overconfident Swimmer).

## 5 Conclusions

(1) Four distinct subtypes of adolescent swimming self-efficacy were identified. Among these, the Fear-Avoidant Swimmer constituted the primary group at 58.6%, reflecting the current reality of inadequate swimming safety knowledge education and safety skills training. The Tentative Swimmer (20.7%) and Assertive Swimmer (14.9%) represent behaviorally prudent groups, while the Overconfident Swimmer at 5.8% indicates individuals with inflated self-efficacy levels, constituting a high-risk group for drowning. These findings provide a practical pathway for targeted interventions in swimming safety education.

(2) The mechanisms associating self-efficacy in swimming with high-risk swimming behaviors are diverse. From a behavioral perspective, self-efficacy presents dual correlational associations on adolescents' swimming practices. As swimming self-efficacy increases, high-risk swimming behaviors tend to rise. Individuals with low or moderate self-efficacy exhibit cautious behavior, actively avoiding high-risk activities. Both Assertive Swimmer and Overconfident Swimmer tend to engage in more high-risk swimming behaviors. The core difference between the two lies in whether their own safety knowledge and practical skills can mitigate the harm risks brought by such high-risk behaviors. Assertive Swimmer has sufficient cognition and skills and has a higher risk buffering ability, while an Overconfident Swimmer has cognitive deviations and weak skills and lacks effective risk buffering; the probability of drowning injury is significantly higher. Therefore, having a high swimming self-efficacy with adequate knowledge and skills will enhance an individual's willingness to participate in water exploration activities, but a complete safety reserve can reduce the risk of injury, while excessive self-efficacy without the support of cognition and skills can easily induce blind risky behaviors.

(3) The mediation analysis revealed that only Water safety skills exerted a mediating effect under excessive self-efficacy, while knowledge and chain mediation failed. This clarifies the underlying pathway of the “double-edged sword” correlational pattern. At the same time, bias-corrected bootstrap mediation tests revealed that the standardized effects of Tentative Swimmer and Assertive Swimmer chain mediation were weak. This suggests that the risk-mitigating effects of traditional, comprehensive water safety education—which relies on a chain of knowledge and skills—are quite limited; therefore, implementing stratified interventions targeting adolescents' swimming self-efficacy is a crucial measure for reducing the risk of drowning.

(4) Cognitive-ability matching is a critical correlate for self-efficacy to display positive behavioral patterns. When enhancing water safety knowledge and skills training, attention must be paid to individuals' potential overconfidence and cognitive biases. A trinity-based training system integrating “belief-cognition-ability” should be established to effectively reduce drowning risks among adolescents.

## 6 Limitations of the study

The data for this study were collected via self-administered questionnaires. The participating students were asked to recall information about themselves, which may have led to recall bias. Because this study employed a cross-sectional design, it was unable to control for several unmeasured confounding factors, which may have introduced bias into the results; therefore, all findings reveal only correlations and do not establish causation. Future research will explore the causal relationships among the variables through longitudinal studies. Meanwhile, there are limitations in the geographical distribution of the research samples. Future research should expand the sample range to include children of different age groups and from different regional backgrounds to enhance the universality of the research conclusions.

## Data Availability

The original contributions presented in the study are included in the article/Supplementary material, further inquiries can be directed to the corresponding author/s.
